# The Outcome and CT Findings of Low-Dose Intensity Modulated Radiation Therapy with SQAP in a Cat with Thymoma

**DOI:** 10.3390/vetsci7040203

**Published:** 2020-12-14

**Authors:** Kenji Kutara, Yohei Mochizuki, Akihiro Ohnishi, Ikki Mitsui, Teppei Kanda, Akihiko Sugiyama, Noritaka Maeta, Kosuke Kobayashi, Yuki Shimizu, Yasuhiko Okamura, Taketoshi Asanuma

**Affiliations:** Faculty of Veterinary Medicine, Okayama University of Science, 1-3 Ikoinooka, Imabari 794-8555, Ehime, Japan; y-mochizuki@vet.ous.ac.jp (Y.M.); a-oonishi@vet.ous.ac.jp (A.O.); i-mitsui@vet.ous.ac.jp (I.M.); t-kanda@vet.ous.ac.jp (T.K.); a-sugiyama@vet.ous.ac.jp (A.S.); n-maeta@vet.ous.ac.jp (N.M.); k-kobayashi@vet.ous.ac.jp (K.K.); y-shimizu@vet.ous.ac.jp (Y.S.); y-okamura@vet.ous.ac.jp (Y.O.); t-asanuma@vet.ous.ac.jp (T.A.)

**Keywords:** conformal radiotherapy, feline, sulfoquinovosyl acyl propanediol, thymoma

## Abstract

A 9-year-old male intact domestic cat weighing 4.6 kg was referred for tachypnea. A large mass was visible in computed tomography (CT) scans of the thoracic cavity. A histopathological evaluation of the mass was consistent with thymoma. The cat was treated with 2 × 8 Gy intensity modulated radiation therapy and sulfoquinovosyl acyl propanediol (SQAP). Post radiation therapy (RT), the tumor structure appeared cystic in the CT, and the tumor volume decreased by approximately 80% after aspiration than that before aspiration. The tumor was removed surgically. RT treatment with SQAP made it possible to treat the thymoma with a low total radiation dose.

## 1. Introduction

Sulfoquinovosyl monoacylglyceride, the lead compound of chemically synthesized sulfoquinovosyl acyl propanediol (SQAP), is a sulfoglycolipid originally isolated from natural sources such as sea urchins [1] and marine algae [2]. SQAP has a radiation sensitization effect on human cancer cells implanted into nude mice [3,4]. The mechanism of the radiation sensitization effect of SQAP may involve the reoxygenation of tumor cells [3]. In several clinical studies, adverse systemic events were not detected after administration of 8 mg/kg or less of SQAP in mice and dogs [5,6,7]. When SQAP was used in radiation therapy (RT), dogs with soft tissue sarcoma and thyroid tumor survived more than one year [5].

In dogs and cats, a thymoma is a common cranial mediastinal tumor; cystic thymomas seem to be the most common form in cats [8]. The metastatic rate has been found to be 20% in cats with cystic thymomas, and thus, a thymoma should be considered a malignant tumor [9]. Surgical excision is the most common treatment [8]; however, a higher disease stage is characterized by a larger tumor and invasion, adherence, or both to surrounding structures, making complete excision difficult to impossible. The potential for postoperative morbidity and mortality and recurrence of larger and invasive tumors is often high [10,11]. Therefore, a different treatment modality is needed for their management. These different treatment modalities include chemotherapy [12] and RT [12,13,14,15,16,17,18]. In veterinary and human medicine, the radiation dose used for thymoma typically ranges between 40 and 60 Gy [11,12,13,14,15,16,17]. This report describes the computed tomographic findings following low-dose RT (16 Gy) using SQAP and easy surgical resection in a case of feline thymoma.

## 2. Case Presentation

A 9-year-old male neutered domestic cat weighing 4.6 kg was referred to Okayama University of Science Veterinary Medical Teaching Hospital (OUS-VMTH) with tachypnea and suspected pleural effusion according to an X-ray and ultrasound examination. Fluid aspiration of the chest had been performed in the referral hospital one month prior. Upon physical examination, cyanosis or abnormality of the skin was not present. A complete blood count and chemistry panel revealed mildly increased hematocrit, blood urea nitrogen, and albumin; the coagulation panel was normal. Thoracic radiography showed an interstitial pattern in both lungs, and the tumor was present in the ventral thoracis. The heart had been displaced caudally and to the right by the mass lesion. Megaesophagus was not apparent. In an ultrasound, the heart showed no morphological abnormality. A mass with cystic characteristics was detected in the precordium; pleural effusion was not shown. Approximately 100 mL of fluid was aspirated from the mass. A cytological evaluation of the fluid performed by a board-certified pathologist revealed that it was dark brown and composed of erythrocytes, small lymphocytes, and macrophages.

**Figure 1:**
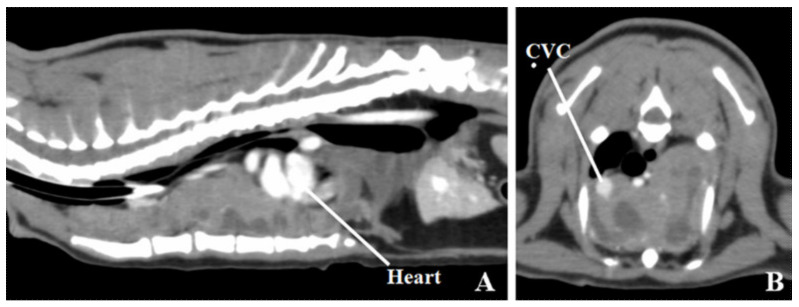
A contrast computed tomography image of the thorax. (**A**) Sagittal image. (**B**) Transverse image at level fifth thoracic vertebra. A large mass is present with contrast-enhancing soft tissue-attenuating regions and noncontrast low-attenuating regions with suspected fluid retention in the ventral thoracic cavity. The heart is displaced caudally and to the right. Arterial vessels in the precordium (brachiocephalic trunk, left subclavian artery) are moved dorsally by the mass. The cranial vena cava (CVC) is moved to the right by the mass.

A whole-body computed tomography (CT) was performed with a 16-slice multidetector CT scanner (Aquilion Lightning, Canon Medical Systems, Otawara, Japan) under anesthesia. Precontrast CT images were collected, followed by contrast CT images after intravenous administration of 2 mL/kg iopamidol (Oypalomin 300, Fuji Pharma Co., Ltd., Tokyo, Japan). A large mass with a contrast-enhancing soft tissue-attenuating and a noncontrast low-attenuating region with suspected cyst was identified in the ventral part of thoracic cavity (Figure 1A,B). Compression from the mass had caused the heart to move right and caudally. The arterial vessels of the precordium (the brachiocephalic trunk and left subclavian artery) were moved dorsally by the mass. The cranial vena cava was moved to the right by the mass (Figure 1B). Vessel-flattening by the mass was not apparent. The cranial sternal and cranial mediastinal lymph nodes were indistinct because the mass filled the anterior mediastinum. The lungs were compressed by the mass, with pulmonary collapse on the ventral aspect. A Tru-cut biopsy was performed. Board-certified pathologists performed a histopathological evaluation and thymoma was diagnosed.

**Figure 2:**
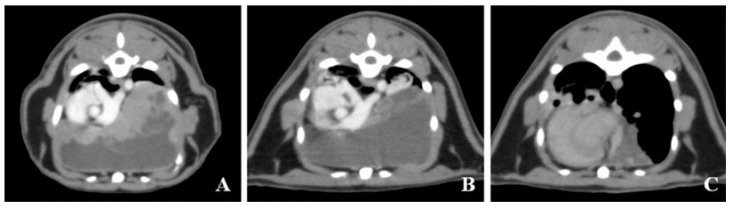
Transverse images of contrast computed tomography (CT) (**A**) preradiation therapy (RT), (**B**) post-RT, and (**C**) post-aspiration at eighth thoracic vertebra level. The pre-RT CT images obtained for treatment planning reveal fluid retention in the tumor, though the fluid is aspirated at initial diagnosis. The volume of tumor is not decreased in post-RT. The tumor structure is changed to cystic post-RT, and the tumor volume is decreased by approximately 80% after aspiration than that before aspiration.

Twenty-six days after the initial CT scanning, the cat was accepted to start RT aimed at decreasing tumor volume before surgery. Pre- and postcontrast CT images were acquired and used for RT planning, immobilization, and positioning in ventral recumbency, using an inflatable mattress (Vac-Lok^TM^ Cushion, Toyo Medic, Tokyo, Japan). The CT images for planning revealed fluid retention in the tumor, even though fluid was aspirated at initial diagnosis (Figure 2A). The radiation plan was devised by the veterinarians who used planning software for contouring and treatment planning (Monaco, Elekta Japan, Tokyo, Japan) using the Monte Carlo equivalent algorithm. Pre- and postcontrast CT images were coregistered prior to contouring. Contouring was performed in precontrast images with 2 mm CT slice thickness. The gross tumor volume (GTV) (target volume margin of RT) was defined as gross tumor on the CT images. The expansion of the clinical target volume margin of RT was not used. The minimum, maximum, and mean dose delivered to the GTV for RT was 14.9, 19, and 16.9 Gy, respectively. The dose-volume histogram constraints for the organs at risk were as follows: the mean doses to the liver, lungs, spine, and heart were 7.6, 9.1, 7.0, and 9.5 Gy, with the minimum doses of 1.2, 1.1, 1.6, and 2.2 Gy, and the maximum doses of 17.3, 17.7, 12.4, and 17.7 Gy, respectively. The isocenter and beam arrangements were determined by the planning target volume locations. RT was delivered by 6 MV photons from a linear accelerator (Elekta Synergy, Canon Medical Systems, Otawara, Japan), using a step and shoot technique. The linear accelerator was equipped with a 160-leaf, 0.5 cm multileaf collimator, which was used to shape the fields to expose the target volume and block the adjacent normal tissue. The cat was administered 8 Gy per fraction, delivered to the total dose of 16 Gy, with one fraction per week for a total of two fractions. Before each RT session, SQAP (4 mg/kg) was injected intravenously as a radiosensitizing agent, with consent from the owner. The use of SQAP was approved by the ethics committee of the OUS-VMTH (approval number, 2020-03). RT was performed 15 min after the injection of the radiosensitizing agent. No radiation toxicity was observed in the patient during the two RT treatment fractions. 

After the second RT session, CT scans were performed. The tumor volume showed little change. However, the content of the tumor changed in the noncontrast low-attenuating region and fluid retention in the tumor was suspected. Most of the contrast-enhancing soft tissue-attenuating region had disappeared in the tumor (Figure 2B). Approximately 220 mL of fluid was aspirated from the mass. A CT scan was repeated after fluid aspiration. Analysis of the CT images on a workstation (VAZE: Pet Communications, Chuo, Osaka, Japan) showed that the tumor volume after aspiration was decreased by approximately 80% than that before aspiration (Figure 2C). The tachypnea went into remission after the CT scanning and fluid aspiration.

**Figure 3:**
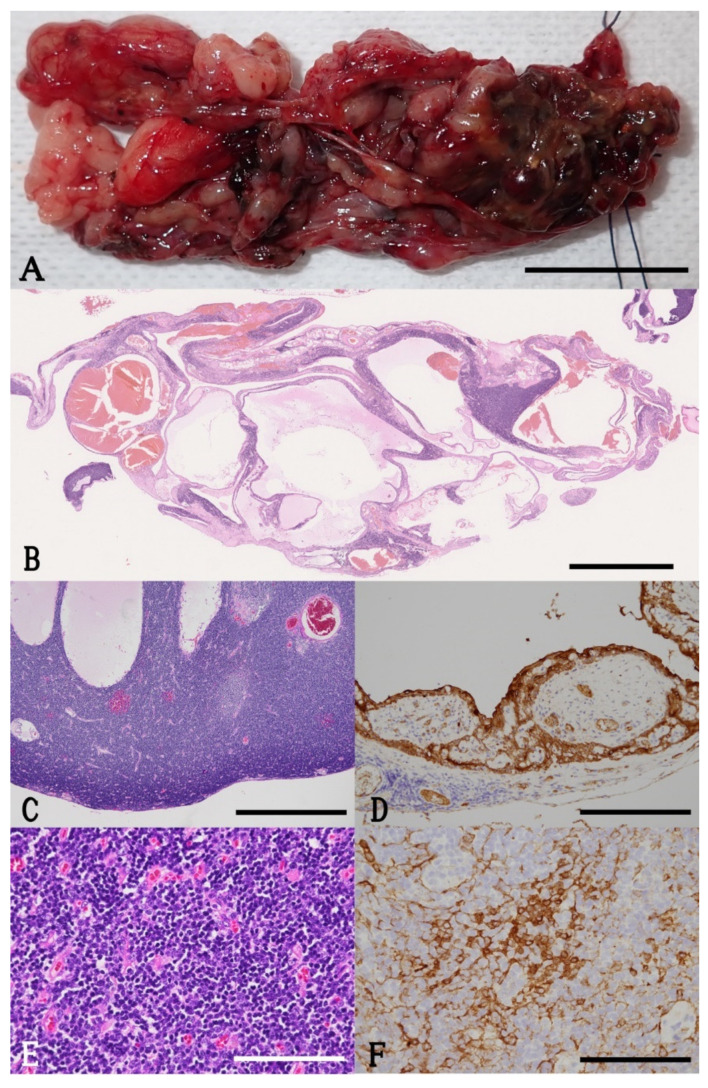
Photographs of the excised tumor. (**A**) Macroscopic view. The removed tumor consists of numerous, pale tan to dark red, variably sized cystic structures and scattered soft nodules. Bar = 2 cm. (**B**) Subgross whole slide image. The tumor is composed of fluid-or-blood-filled cysts and solid neoplastic tissue. Hematoxylin and eosin stain; bar = 3 mm. (**C**) Photomicrograph ×40. The neoplastic tissue shows medullary differentiation manifested by darker periphery and lighter center, resembling thymic cortex and medulla. Cystic spaces are also present. Hematoxylin and eosin stain; bar = 1 mm. (**D**) Photomicrograph ×200. The cysts within the lesion are lined by pleomorphic cells, which are positive for cytokeratin AE1/AE3. Immunohistochemistry; bar = 200 µm. (**E**) Photomicrograph ×400. The neoplastic tissue is composed of dense sheets of mature small lymphocytes (reactive component) with scattered, ovoid, stellate, or elongated cells (neoplastic component). Hematoxylin and eosin stain; bar = 100 µm. (**F**) Photomicrograph ×400. Neoplastic cells are positive for cytokeratin AE1/AE3. Immunohistochemistry; bar = 100 µm.

Fourteen days after the last CT scan, the cat was accepted for surgery to remove the tumor. A histopathological evaluation was performed by the pathologists. The mass was composed of dense sheets of mature small T lymphocytes showing medullary differentiation with a few, scattered, ovoid, stellate, or elongated cells that were positive for cytokeratin AE1/AE3 (Figure 3). Scattered within the mass were variably sized cysts filled with proteinaceous fluid and blood. These cysts were lined by keratinized or nonkeratinized pleomorphic cells. These histologic features are consistent with type B1 thymoma.

More than 160 days after the surgery, the general condition of the cat was stable. No radiation toxicity was observed in the at-risk organs, including the heart and lungs.

## 3. Discussion

Based on our review of the literature, this is the first report describing the clinical management, low-dose intensity modulated radiation therapy (IMRT) (total dose: 16 Gy) using SQAP, aspiration, and surgical resection for feline thymoma. The tumor volume had not changed after low-dose IMRT using SQAP. However, the tumor structure changed to cystic, and after aspiration, the tumor volume was decreased by approximately 80% than that before aspiration. Furthermore, the tumor was easily removed in surgery. Sulfoquinovosyl monoacylglyceride, the lead compound of chemically synthesized SQAP, is sulfoglycolipids originally isolated from natural sources such as sea urchins [1] and marine algae [2]. As SQAP led to increased tumor oxygenation in mice, it was considered that the radiosensitizing effect of this agent was to enhance the oxygen-dependent effects of radiotherapy in tumors [3]. In one clinical study [5], tumor volume was found to decrease when SQAP was used with RT for dogs with thyroid tumor and hemangiosarcoma, while dogs with soft tissue sarcoma and thyroid tumor survived for more than one year. In dogs, high-dose SQAP administration (>8 mg/kg) results in the adverse effect of angialgia [5]. However, adverse systemic events were not detected after the administration of 8 mg/kg or less in mice and dogs [5,6,7]. In these studies, the recommended dose of SQAP was 2–4 mg/kg. In the current study, we used an SQAP dose of 4 mg/kg and detected no adverse systemic events. Nevertheless, large-scale studies are needed to validate the safety of systemic administration of SQAP in cats.

In the present study, the total RT dose was decided as 16 Gy as we could not control the radiation doses to the lungs, heart, and liver. Lung toxicity is one of the most common concerns, and a dose-limiting toxicity for thoracic irradiation [13,19]. In human medicine, a recent review found that >20% risk of radiation pneumonitis is associated with a mean lung dose of >20 Gy [13,19]. In veterinary and human medicine, the radiation dose for thymoma used usually ranges between 40 and 60 Gy [12,13,14,15,16,17,18]. Due to the size of the tumor in the current study, it was impossible to achieve a mean lung dose of >20 Gy with a conventional radiation dose for thymoma even if IMRT was used. Furthermore, we decided on the total RT dose of 16 Gy after having considered the movement of the lungs and heart due to breathing.

In the current study, we observed that the structure of the tumor changed to cyst-like with a severe decrease in tumor parenchyma after RT, and that after aspiration, the tumor volume was decreased by approximately 80% than that before aspiration. In previous studies of feline thymoma, RT outcomes were that the thymoma size decreased, and a cystic finding remained [8,17]. In this case, the thymoma had remarkable fluid retention and a severe decrease in tumor parenchyma. Thus, it is possible that the atrophy of the tumor parenchyma, caused by RT, and fluid retention in the tumor occurred concurrently. When treating animals with RT using SQAP, large-scale studies are necessary to determine whether cases of other thymoma types show similar findings.

In conclusion, RT using SQAP may enable the treatment of feline thymoma with a low total radiation dose. Additional studies are necessary to make SQAP with IMRT a standard treatment for thymoma.
